# The complete chloroplast genome sequence of *Machilus robusta* W. W. Smith (Lauraceae) from Jiangxi Province, China

**DOI:** 10.1080/23802359.2021.1934160

**Published:** 2021-06-07

**Authors:** Yanfang Wu, Yongjie Zheng, Xinliang Liu, Xiaoying Dai, Shengxing Li, Haining Xu

**Affiliations:** Camphor Engineering Technology Research Center for National Forestry and Grassland Administration, Jiangxi Academy of Forestry, Nanchang, China

**Keywords:** Complete chloroplast genome, Lauraceae, *Machilus robusta* W. W. Smith, phylogeny

## Abstract

*Machilus robusta* W. W. Smith is an evergreen plant distributed in the Yangtze River Basin and the south regions of China. Here we analyzed the complete chloroplast (cp) genome sequence of *M. robusta* to determine its structure and evolutionary relationship to other Lauraceae. The cp genome is 152,737 bp in length and has an overall GC content of 39.2% The genome includes a large single-copy (LSC) region of 93,706 bp and a small single-copy (SSC) region of 18,885 bp, and these are separated by a pair of inverted repeats (IRs) of 20,073 bp. The cp genome contains 128 genes, including 83 protein-coding, 37 tRNAs, and 8 rRNAs. Phylogenetic analysis based on complete cp genome sequences fully resolved *M. robusta* in a clade with *M. balansae.* This work provides new molecular data for evolutionary studies of the Lauraceae.

*Machilus robusta*, an evergreen plant of the genus classified in the Lauraceae, is mainly distributed in the Yangtze River Basin and the south regions of China, with Guangdong, Guangxi, Guizhou, Hainan, Xizang, Yunnan as the main growing area. Its leaves are rich in bioactive metabolites with diverse structures, and are known to relaxmuscles, promoteblood circulation, reduceswelling, and relieve pain (Liu et al. 2007). The plant is also widely used as a folk herb for the treatment of gastric dullness, vomiting, and diarrhea in China (Li et al. 2011; Bu et al. 2013). To date, only genetic marker data are publicly available for *M. robusta*. In this study, the *M. robusta* cp genome was sequenced and assembled to document its plastid chromosomal content and structure, as well as confirm its relationship to other Lauraceae.

Fresh leaves of *M. robusta* were collected from Jiangxi Academy of Forestry, Nanchang, Jiangxi, China (28°44′41″N, 115°48′37″E). The voucher specimen was deposited in the laboratory of Camphor Engineering Technology Research Center for National Forestry and Grassland Administration (Yanfang Wu, yanfangwu2012@163.com) under accession number of WYF202001. Genomic DNA was extracted using the DNeasy plant mini kit (Qiagen). Paired-end reads were generated by using the Illumina NovaSeq system (Illumina, San Diego, CA). In total, ∼1.4 Gb of raw data (9,223,296 reads) were obtained. Quality control was performed to remove adapters and low-quality reads using fastp (Chen et al. 2018). The cp genome was *de novo* assembled by NOVOPlasty (Dierckxsens et al. 2017; Wang et al. 2018) and annotated by GeSeq (Tillich et al. 2017).

The complete cp genome sequence of *M. robusta* is 152,737 bp in length, and contains a large single-copy region (LSC) of 93,706 bp, a small single-copy region (SSC) of 18,885 bp, and a pair of inverted repeats (IR) regions of 20,073 bp. A total of 128 genes were annotated, including 83 protein-coding genes, 37 tRNAs, and 8 rRNAs. The GC content of the cp genome is 39.2%. To reveal the phylogenetic position of *M. robusta* with other members in Lauraceae, a phylogenetic analysis was performed based on 37 complete cp genomes, and two taxa from the Calycanthaceae, *Calycanthus chinensis* and *Chimonanthus nitens* to serve as outgroups. The sequences were aligned by MAFFT v7.271 (Katoh and Standley 2013). The maximum likelihood (ML) bootstrap analysis with 1000 replicates was performed using IQ-TREE v1.6.12 (Minh et al. 2020). TVM + F+R3 was selected as the best-fit model according to the built-in ModelFinder. The phylogenetic tree showed that *M. robusta* is closely related to *M. balansae* (Figure 1). *Machilus* was fully resolved in a sister position to the genus *Phoebe* in Lauraceae. These results were similar to those presented by Kong et al. (2014) based on matK sequence analysis and Chen et al. (2017) based on cp gene analysis. The cp genome sequence of *M. robusta* provides new molecular data for evolutionary studies of Lauraceae.

**Figure 1.**
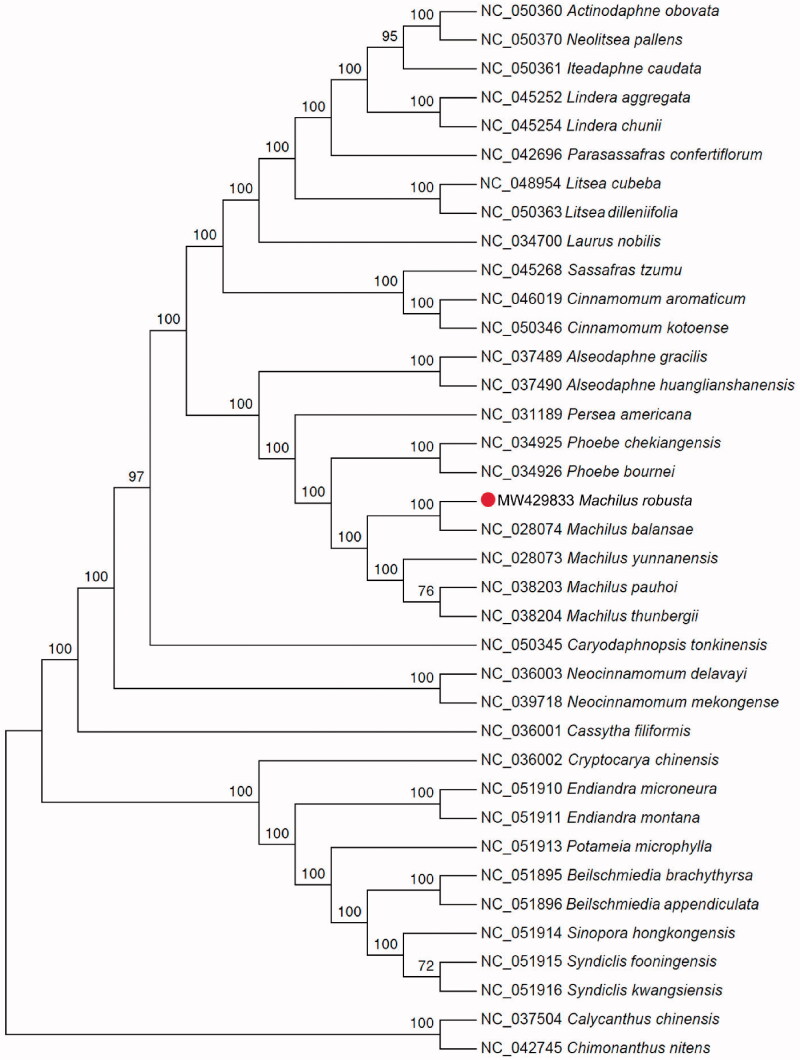
Maximum likelihood phylogenetic tree based on the complete chloroplast genome sequences of 35 plant species from Lauraceae and two outgroup plant species from the Calycanthaceae.

## Data Availability

The genome sequence data that support the findings of this study are openly available in GenBank of NCBI at [https://www.ncbi.nlm.nih.gov] (https://www.ncbi.nlm.nih.gov/) under the accession no. MW429833. The associated BioProject, SRA, and Bio-Sample numbers are PRJNA714487, SRR13962180, and SAMN18310375 respectively.
